# First trimester medication abortion practice in the United States and Canada

**DOI:** 10.1371/journal.pone.0186487

**Published:** 2017-10-12

**Authors:** Heidi E. Jones, Katharine O’Connell White, Wendy V. Norman, Edith Guilbert, E. Steve Lichtenberg, Maureen Paul

**Affiliations:** 1 Dept. of Epidemiology & Biostatistics, CUNY School of Public Health, New York, New York, United States of America; 2 Dept. of Obstetrics & Gynecology, Baystate Medical Center, Springfield, Massachusetts, United States of America; 3 Dept. of Obstetrics & Gynecology, Boston University/Boston Medical Center, Boston, Massachusetts, United States of America; 4 University of British Columbia, Vancouver, British Columbia, Canada; 5 Institut National de Santé Publique du Québec, Québec, Canada; 6 Family Planning Medical Associates Medical Group, Chicago, Illinois, United States of America; 7 Beth Israel Deaconess Medical Center, Harvard Medical School, Boston, Massachusetts, United States of America; National Academy of Medical Sciences, NEPAL

## Abstract

We conducted a cross-sectional survey of abortion facilities from professional networks in the United States (US, n = 703) and Canada (n = 94) to estimate the prevalence of medication abortion practices in these settings and to look at regional differences. Administrators responded to questions on gestational limits, while up to five clinicians per facility reported on 2012 medication abortion practice. At the time of fielding, mifepristone was not approved in Canada. 383 (54.5%) US and 78 (83.0%) Canadian facilities participated. In the US, 95.3% offered first trimester medication abortion compared to 25.6% in Canada. While 100% of providers were physicians in Canada, just under half (49.4%) were advanced practice clinicians in the US, which was more common in Eastern and Western states. All Canadian providers used misoprostol; 85.3% with methotrexate. 91.4% of US providers used 200 mg of mifepristone and 800 mcg of misoprostol, with 96.7% reporting home misoprostol administration. More than three-quarters of providers in both countries required an in-person follow-up visit, generally with ultrasound. 87.7% of US providers routinely prescribed antibiotics compared to 26.2% in Canada. Nonsteroidal anti-inflammatory drugs were the most commonly reported analgesic, with regional variation in opioid narcotic prescription. In conclusion, medication abortion practice follows evidence-based guidelines in the US and Canada. Efforts to update practice based on the latest evidence for reducing in-person visits and increasing provision by advanced practice clinicians could strengthen these services and reduce barriers to access. Research is needed on optimal antibiotic and analgesic use.

## Introduction

Medication abortion ranks among the most important advances in women’s reproductive health in the last several decades. This non-invasive option for pregnancy termination is convenient, effective, and safe.[1] Currently registered in more than 60 countries worldwide,[2] mifepristone is the gold standard for early medication abortion. Evidence-based regimens using mifepristone in combination with a prostaglandin carry success rates up to 99% for early pregnancy termination with rare occurrence of serious adverse events.[3, 4] In countries where mifepristone is not available, methotrexate combined with misoprostol or misoprostol alone remain important alternatives.

While abortion is common in the US and Canada, with approximately one in three women having an abortion during her lifetime in both countries,[5, 6] abortion practice, especially medication abortion, may differ by region. The US Food and Drug Administration (FDA) approved mifepristone with misoprostol for medication abortion in 2000. Subsequently, the proportion of early abortions (≤8 weeks gestation) that were medication abortions rose from 4.6% in 2001 [7] to 30.8% in 2012.[8] Health Canada approved mifepristone for medication abortion in 2015. Prior to this approval, Canadian providers used methotrexate-misoprostol or misoprostol alone. Although medication abortion in Canada accounted for only 4% of hospital-based abortions in 2012,[9] it likely will increase with this recent approval of mifepristone.

Given the magnitude of medication abortion services in the US and Canada, understanding the extent to which practice follows evidence-based guidelines is important. Further, regional variation in practice may indicate areas that require additional research to identify best practice and may affect abortion access. We therefore compare first trimester medication abortion practice for the US and Canada and, within the US, by region.

## Materials and methods

We conducted a cross-sectional survey of abortion providers in order to examine 2012 abortion practice in the US and Canada. Previously, we conducted national surveys of a large professional network of abortion providers in the US in 1997 [10] and 2002, [11–13] and in British Columbia, Canada in 2011.[14, 15] For the current study, we identified 703 clinical sites in the US and 94 in Canada through websites, such as www.abortion.com, and professional networks, such as Planned Parenthood. In the US, as we did not review telephone directories or call private physicians’ offices or hospitals, our sample is not a complete census. The estimate of sites performing abortions in the US in 2011 is 329 abortion clinics, 510 other clinics and 881 hospitals or private physicians offices.[16] Most high volume clinics were included in the current sample, given the inclusion of professional networks of abortion providers. Additionally, we included hospitals that participated in the Ryan Residency Training Program in Abortion and Family Planning and the Fellowship in Family Planning, as key sites of training for the next generation of providers. Thus, our sampling frame represents the majority of abortion clinics and the major abortion-related academic training programs in US residency and post-residency programs, but does not represent private physician’s offices or hospital providers, which provided an estimated 5% of abortions in 2011.[16] In Canada, we could not identify an estimate of all abortion sites, and all attempts were made to include a census. However, it is likely that some private physician’s offices were missed.

We updated the 2002 questionnaires [11–13] in order to include questions on new laws and evidence-based guidelines. A few questions, such as those on medication abortion drug regimens, differed by country. A certified translator translated Canadian questionnaires into French for fielding in Québec. The study was approved by ethical committees at the City University of New York and the University of British Columbia, and deemed exempt from review at Beth Israel Deaconess Medical Center and Baystate Medical Center. As the survey was self-administered, participants read informed consent information prior to participation.

Fielding occurred from June through December of 2013. Three types of questionnaires were sent by mail or electronically to each site: one to be completed by the facility administrator, a second by up to five surgical abortion providers, and a third by up to five medication abortion providers who performed the most abortions at their site in 2012. Mailed packages included postage-paid addressed return envelopes. We contacted non-respondents during two rounds of phone calls and/or emails to encourage participation.

Some administrators completed a single survey for a network of facilities. We created a frequency weight for the number of individual sites for which they responded that performed medication abortions; we present weighted responses with the site as the unit of analysis for all facility-level results. Clinicians who provided medication abortions at multiple facilities were instructed to complete one survey to represent usual practice. A few clinicians completed multiple surveys; we included responses for the site at which they reported the greatest number of medication abortions. We analyzed each unique clinician as an independent observation for clinician-level results and did not compare responses between clinicians working at the same facility to allow for practice variation within a facility. We included three clinicians in these analyses for whom no facility-level data were available.

Administrators reported the total number of abortions performed in 2012 and the gestational limit for medication abortions at their site(s). Clinicians reported personal demographic characteristics, clinical specialty, and routine practice for use of ultrasound, medication regimen, use of analgesics and antibiotics and follow-up procedures. We compared medication abortion practice by country, and, within the US, by region using a chi-squared or Fisher’s exact test when cell counts were small in SPSS version 23 (IBM, Armonk, NY, USA). We used a critical alpha of 0.001 to adjust for multiple comparisons. Regional differences within Canada are described elsewhere.[17]

## Results

Of the 703 clinical sites identified in the US, 383 (54.5%) participated; 223 administrators responded for 381 facilities, and for two facilities only clinicians responded. Of the 94 clinical sites identified in Canada, 78 (83.0%) participated (Table 1); 74 administrators responded for 77 facilities, and for one facility only one clinician responded. In Canada, every province was represented, except for Prince Edward Island, for which no clinical site was identified.[18] Participating sites provided an estimated 419,159 first and second trimester abortions in the US and 75,650 in Canada in 2012. In the US, the majority (n = 363, 95.3%) of sites offering abortion services provided first trimester medication abortion compared to 25.6% (n = 20) in Canada. Sites which provided medication abortions provided an estimated 135,503 first trimester medication abortions in the US and 2,706 in Canada in 2012, representing 35.4% and 3.8% respectively of all first trimester abortions reported across all sites and 36.1% and 10.3% of first trimester abortions reported by sites offering medication abortion.

**Table 1:** Facility and clinician level characteristics of medication abortion providers by region and country, cross-sectional survey of 2012 abortion practice in the US and Canada.

	Regions within the United States (US)**					Country		
	East	South	Midwest	West	p-value	US	Canada	p-value
**Facility-level results**								
Clinical sites identified	171	188	112	232	-	703	94	-
Clinical sites participated, n (%)	116 (67.8)	55 (29.3)	46 (41.1)	166 (71.6)†	<0.001	383 (54.4)†	78 (83.0)†	<0.001
Clinical sites offer medication abortion, n (%)	115 (99.1)	49 (89.1)	41 (89.1)	158 (96.3)	<0.001	363 (95.3)	20 (25.6)	<0.001
Type of facility, n (%)								
Private office	12 (10.4)	22 (44.9)	11 (26.8)	44 (27.8)	<0.001	89 (24.5)	4 (20.0)	<0.001
Ambulatory health center	86 (74.8)	19 (38.8)	25 (61.0)	104 (65.8)		234 (64.5)	7 (35.0)	
Hospital-affiliated	17 (14.8)	8 (16.4)	5 (12.2)	10 (6.3)		40 (11.0)	9 (45.0)	
Latest gestational age in LMP for medication abortions (%)††:								
49 days	1 (0.9)	2 (4.1)	4 (10.0)	1 (0.6)	<0.001	8 (2.2)	15 (83.3)	<0.001
56 days	6 (5.2)	7 (14.3)	0 (0.0)	3 (1.9)		16 (4.4)	3 (16.7)	
63 days	96 (83.5)	38 (77.6)	28 (70.0)	147 (93.0)		309 (85.4)	0 (0.0)	
70 days	9 (7.8)	1 (2.0)	5 (12.5)	6 (3.8)		21 (5.8)	0 (0.0)	
98 days	3 (2.6)	1 (2.0)	3 (7.5)	1 (0.6)		8 (2.2)	0 (0.0)	
**Clinician-level results**								
Medication abortion clinicians participated, n*	92	45	46	165	-	348	62	-
Age of clinician in years, n (%)								
24–30	4 (4.5)	1 (2.3)	0 (0.0)	12 (7.4)	<0.001	17 (5.0)	1 (1.6)	0.214
31–40	20 (22.5)	6 (13.6)	14 (31.8)	54 (33.3)		94 (27.7)	19 (31.1)	
41–50	25 (28.1)	8 (18.2)	15 (34.1)	34 (21.0)		82 (24.2)	15 (24.6)	
51–60	18 (20.2)	6 (13.6)	3 (6.8)	38 (23.5)		65 (19.2)	17 (27.9)	
61–70	18 (20.2)	14 (31.8)	7 (15.9)	24 (14.8)		63 (18.6)	9 (14.8)	
71–89	4 (4.5)	9 (20.5)	5 (11.4)	0 (0.0)		18 (5.3)	0 (0.0)	
Female, n (%)	74 (80.4)	20 (44.4)	34 (73.9)	146 (88.5)	<0.001	274 (78.7)	49 (80.3)	0.778
Clinical degree, n (%)								
Medical doctor/doctor osteopathic medicine	40 (46.0)	40 (93.0)	39 (90.7)	46 (30.1)	<0.001	165 (50.6)	62 (100.0)	<0.001
Nurse practitioner	27 (31.0)	1 (2.3)	2 (4.7)	65 (42.5)		95 (29.1)	0 (0.0)	
Physician assistant	9 (10.3)	2 (4.7)	0 (0.0)	10 (6.5)		45 (13.8)	0 (0.0)	
Certified nurse midwife	11 (12.6)	0 (0.0)	2 (4.7)	32 (20.9)		21 (6.4)	0 (0.0)	
Provide first trimester surgical abortions, n (%)	45 (48.9)	40 (88.9)	39 (84.8)	64 (38.8)	<0.001	188 (54.0)	54 (88.5)	<0.001

^†^ Two facilities in the Western US and two in Canada did not complete administrative data;

^††^ One facility in Midwest, US and two in Canada are missing this data.

* Clinician sample size varies depending on the number of responses with missing data for a given measure, ranging from 326–348 in the US (87–92 in the East, 43–45 in the South, 43–46 in the Midwest, 153–165 in the West) and 61–62 in Canada /

** East = Connecticut, Delaware, Massachusetts, Maine, New Hampshire, New Jersey, New York, Pennsylvania, Rhode Island, Vermont; South = Alabama, Arkansas, Washington D.C., Florida, Georgia, Kentucky, Louisiana, Maryland, Mississippi, North Carolina, Oklahoma, Puerto Rico, South Carolina, Tennessee, Texas, Virginia, West Virginia; Midwest = Iowa, Illinois, Indiana, Kansas, Michigan, Minnesota, Missouri, Nebraska, North Dakota, Ohio, South Dakota, Wisconsin; West = Alaska, Arizona, California, Colorado, Hawaii, Idaho, Montana, New Mexico, Nevada, Oregon, Utah, Washington, Wyoming.

Among sites providing first trimester medication abortion, 33.3% did not provide surgical abortions in the US, compared to only one out of 20 sites (5.0%) in Canada. Sites that offered medication abortion in the US were primarily ambulatory health centers (64.5%), while in Canada, ambulatory health centers (35.0%) and private offices (20.0%) together comprised 55.0% of sites (Table 1).

### Gestational age limit

In the US, most facilities (85.4%) offered medication abortion through 63 days last menstrual period (LMP). More facilities in the South stopped at 49 or 56 days LMP (18.4%) than in other regions (10.0% in the Midwest, 6.1% in the East and 2.5% in the West, p<0.001). In Canada, most sites offered medication abortion through 49 days LMP (83.3%).

### Clinician characteristics

We received 348 surveys in the US and 62 in Canada from medication abortion clinicians. Only 16 participating facilities that provided medication abortion did not provide a medication abortion clinician survey. While provider age did not differ by country, within the US, providers in the South tended to be older with more than half (52.3%) over the age of 60, compared to less than a quarter (23.9%) for the US as a whole (Table 1). In Canada, 100% of providers were physicians, as required by law. In the US, about half of providers (49.4%) who responded to this question (n = 326, 93.6%) were advanced practice clinicians; this proportion was higher in the East (54.0%) and West (69.9%). This pattern was reflected in the provision of first trimester surgical abortions; while most providers in Canada (88.5%) also provided first trimester surgical abortions, slightly more than half (54.0%, p<0.001) of US providers did.

### Medication abortion regimens

Almost all US providers reported using 200 mg of mifepristone and 800 mcg of misoprostol (91.4%); most (81.8%) provided misoprostol for use 24 to 48 hours after mifepristone. Only 2.4% of providers used 600 mg of mifepristone and 400 mcg of misoprostol; most (n = 7, 87.5%) provided misoprostol for use 48 hours after mifepristone. Most US providers reported administration of mifepristone at the facility (92.9%), and buccal (84.7%) administration of misoprostol at home (96.7%, Table 2). The majority of misoprostol administration at the facility was oral administration (8/14, 57.1%).

**Table 2:** Clinician reports on medication abortion practice by region and country, cross-sectional survey of 2012 abortion practice in the US and Canada.

	Regions within the United States (US)					Country		
	East (n = 92)†	South (n = 45)†	Midwest (n = 46)†	West (n = 165)†	p-value	US (n = 348)†	Canada (n = 62)†	p-value
Mifepristone dose, n (%)								
200 mg	90 (100.0)	44 (100.0)	35 (81.4)	162 (100.0)	P<0.001	331 (97.6)	na	
600 mg	0 (0.0)	0 (0.0)	8 (18.6)	0 (0.0)		8 (2.4)		
Other	0 (0.0)	0 (0.0)	0 (0.0)	0 (0.0)		0 (0.0)		
Where mifepristone is taken, n (%)								
At home	8 (8.9)	5 (11.6)	3 (7.0)	8 (5.0)	0.411	24 (7.1)	na	
At medical facility	82 (91.1)	38 (88.4)	40 (93.0)	153 (85.0)		313 (92.9)		
Methotrexate dose, n (%)								
50 mg/m^2^ body surface area	na						42 (68.9)	na
50 mg fixed dose							10 (16.4)	
Don’t use methotrexate							9 (14.8)	
Initial misoprostol dose, n (%)								
400 mcg	4 (4.4)	1 (2.3)	10 (23.3)	10 (6.3)	0.001	25 (7.4)	4 (6.6)	0.933
800 mcg	86 (95.6)	43 (97.7)	33 (76.7)	145 (91.2)		307 (91.4)	57 (93.4)	
Other	0 (0.0)	0 (0.0)	0 (0.0)	4 (2.5)		4 (1.2)	0 (0.0)	
Misoprostol route, n (%)								
Vaginal	10 (11.1)	9 (20.5)	3 (7.0)	7 (4.3)	<0.001	29 (8.5)	47 (77.0)	<0.001
Oral (swallowed)	0 (0.0)	0 (0.0)	10 (23.3)	1 (0.6)		11 (3.2)	0 (0.0)	
Sublingual (under tongue)	0 (0.0)	1 (2.3)	0 (0.0)	2 (1.2)		3 (0;9)	6 (9.8)	
Buccal (between cheek and gum)	80 (89.1)	31 (70.5)	29 (67.4)	148 (90.8)		288 (84.7)	8 (13.1)	
Other	0 (0.0)	3 (6.8)	1 (2.3)	5 (3.1)		9 (2.6)	0 (0.0)	
Where misoprostol is taken, n (%)								
At home	89 (100.0)	43 (97.7)	32 (76.2)	163 (100.0)	<0.001	327 (96.7)	58 (95.1)	0.458
At medical facility	0 (0.0)	1 (2.3)	10 (23.8)	0 (0.0)		11 (3.3)	3 (4.9)	
Repeat dose of misoprostol, n (%)								
Part of take-home medications	2 (2.2)	3 (6.8)	0 (0.0)	2 (1.2)	0.161	7 (2.1)	57 (93.4)	<0.001
Given only as needed	85 (94.4)	39 (88.6)	41 (95.3)	159 (97.5)		324 (95.3)	3 (4.9)	
Never given	3 (3.3)	2 (4.5)	2 (4.7)	2 (1.2)		9 (2.6)	1 (1.6)	
Provide antibiotic to every patient, n (%)	77 (85.6)	29 (65.9)	40 (90.9)	153 (93.9)	<0.001	299 (87.7)	16 (26.2)	<0.001
Offer by telemedicine, n (%)	1 (1.1)	0 (0.0)	8 (18.2)	2 (1.2)	<0.001	11 (3.2)	4 (6.6)	0.260
Require in-person follow up, n (%)	81 (88.0)	41 (93.2)	34 (75.6)	126 (76.4)	0.015	282 (81.5)	47 (77.0)	0.415
Require ultrasound before procedure, n (%)	89 (98.9)	44 (100.0)	43 (97.7)	158 (98.8)	0.805	334 (98.8)	56 (91.8)	0.006
Require follow up with ultrasound, n (%)	58 (63.7)	37 (84.1)	22 (48.9)	114 (69.5)	0.004	231 (67.2)	41 (67.2)	0.992

^†^ The total sample size (n) varies depending on the number of responses with missing data for a given measure, ranging from 336–348 for the US (89–92 in the East, 43–45 in the South, 42–46 in the Midwest, 159–165 in the West) and 61–62 for Canada./ na = not applicable: methotrexate is only used in Canada not in the US, and misoprostol was only available in the US and not Canada during the survey fielding.

In Canada, 14.8% of providers used misoprostol alone, while the majority used methotrexate with misoprostol, most commonly 800 mcg (91.8%). Of those who used methotrexate-misoprostol, all provided misoprostol within 7 days of methotrexate, generally 4–6 days (34.6%), 1–2 days (23.1%) or 3 days (21.2%).

### Analgesics and antibiotics

Only one provider in the US (0.3%) and six in Canada (9.8%) did not provide pain medication routinely. The most common analgesics dispensed routinely were nonsteroidal anti-inflammatory drugs (NSAIDS, 76.2% of US providers and 73.8% in Canada). Opioid narcotics were routinely prescribed by 56.3% of US providers compared to 13.1% of Canadian providers (p<0.001). This practice was more commonly reported in the Midwest (68.2%) and the West (67.5%) than in the East (36.7%) or South (43.2%, p<0.001).

In the US, 87.7% of providers routinely prescribed antibiotics for medication abortions, while only 26.2% of Canadian providers did so (Table 2). The most common regimens in the US were 7 days of doxycycline (64.2%) or a single dose of azithromycin (29.1%), while in Canada, the most common regimens were one dose of metronidazole (31.2%), one day of doxycycline (18.8%) or one dose of azithromycin (18.8%).

### Follow-up procedures

In both countries, more than three-quarters of providers (81.5% and 77.0%, respectively) required an in-person follow-up visit. Most providers (95.1%, US; 73.8%, Canada) used ultrasound as the most common method for assessing successful pregnancy termination. Routine use of serial hCGs was more common in Canada (21.3%) than in the US (2.6%, p<0.001). However, 11.1% of providers in the Midwest reported using serial hCGs compared to 0.0–4.7% in other regions (p = 0.001).

### Eligibility

In the US, most (98.2%) providers offered medication abortions to people less than 18 years of age, while in Canada 75.0% of providers reported doing so (p<0.001). In the US, nearly all (98.8%) providers required a pre-procedure ultrasound compared to 91.8% in Canada (p = 0.006). About a third of providers in both countries (38.4% in the US and 34.4% in Canada) offered medication abortion when no gestational sac was seen on ultrasound, and 86.9% in the US and 80.0% in Canada when no yolk sac or fetal pole was seen, but gestational sac was present. Very few providers (3.2% in the US and 6.6% in Canada) reported provision of medication abortion by telemedicine.

## Discussion

This 2012 survey of US and Canadian abortion providers revealed continued uptake of medication abortion by US providers and strong adherence to evidence-based guidelines.[19–22] Most clinics (95%) in the US provided medication abortions in 2012, an increase from 87% from 2001,[11] with the vast majority using the evidence-based regimen of mifepristone 200 mg combined with misoprostol 800 mcg. Compared to 2001 in which 92% of misoprostol administration was vaginal,[11] buccal administration was most common, aligning with a 2006 guideline change.[23] A higher proportion of clinics (85%) provided medication abortion through 63 days LMP compared to 65% in 2001;[11] 8% offered medication abortion past 63 days LMP, reflecting emerging evidence of efficacy at later gestational ages.[24] In Canada, in the absence of mifepristone availability, the proportion of first trimester abortions accomplished by non-surgical means and the proportion of facilities offering medication abortion were significantly lower than in the US, and, as per Canadian guidelines,[21] went up to 49–56 days LMP.

The practice patterns of US and Canadian providers varied in areas where evidence was insufficient to recommend best approaches. While NSAIDs were the most common analgesic used, more than half of US providers routinely prescribed opioid narcotics. Except for research supporting superior efficacy of ibuprofen over acetaminophen for medication abortion,[25] robust data comparing analgesic regimens are lacking. US providers also were more likely than their Canadian counterparts to prescribe antibiotics routinely and to favor full treatment doses. Routine use of antibiotics increased in the US when Planned Parenthood Federation of America, in response to emerging cases of rare but serious post-abortal infections, modified its practice guidelines to require buccal rather than vaginal administration of misoprostol and universal antibiotic treatment. Although data published in 2009 revealed a decrease in the rate of serious infections following this dual intervention,[26] later research cast doubt on whether antibiotics contributed to the improvement.[23] Given lack of convincing evidence and the risk of side effects and antibiotic resistance, guidelines of the National Abortion Federation (NAF),[20] the American College of Obstetricians and Gynecologists (ACOG),[19] the Society of Family Planning,[19] and the World Health Organization [22] do not recommend routine antibiotic prophylaxis for medication abortion.

Scientific and policy advances, including Health Canada’s approval of mifepristone for medication abortion, present important opportunities to expand access to abortion care. The degree to which this occurs, however, will depend on many factors, including regulations, health systems, and the willingness of providers to adopt new practices. In our survey, most providers permitted home use of misoprostol, but nearly all required pre-procedure sonography and most mandated routine in-person follow-up visits to assess completion of abortion. Accumulated evidence supports alternative approaches that would reduce or eliminate in-person visits for most patients, [27, 28]. In fact, ACOG 2014 [19] and NAF 2016 [29] guidelines include other modalities of clinical evalution to confirm gestational age prior to medical abortion, and state that in-clinic follow-up visits are not always needed. Expansion of telemedicine services, which was reported rarely in our sample, has the potential to increase abortion access in areas lacking abortion providers, as also noted in ACOG 2014 guidelines [19], provided that growing legal restrictions on its use can be successfully challenged. Since 2012, the number of US states with laws prohibiting the use of telemedicine for medication abortion has risen from 7 to 18; like many other abortion restrictions, these laws predominate in the South and Midwest.[30] Workforce disparities also emerged in these regions. More than half of the medication providers in the Southern US were over age 60, and provision by advanced practice clinicians was significantly less common in the South and Midwest than in other areas of the US. Advanced practice clinicians can safely provide first trimester abortions [31] and could reduce gaps in the distribution of the medical workforce, provided laws or labeling restrictions do not prohibit them from service provision.

This study has limitations. The sample may not be representative. However, known professional networks and training sites were included in the sample and the response rate was reasonable (54.4% in the US) to robust (83.0%) in Canada. Further, these sites provided an estimated 90% of all abortions in Canada [9] and 40% in the US,[16] thus, representing a sizable proportion of abortion practice. Given the lower response rates in the Southern (29%) and Midwestern (41%) US, the findings from these regions may be biased and should be interpreted cautiously. Anywhere from one to five clinicians per facility responded to the clinician-level questionnaire, and a number of clinicians worked at more than one facility. We did not adjust for clustering at the facility level, given the complexity of this network. As such, the current analysis does not document the extent to which clinicians’ choices for procedures are affected by facility-level guidelines or culture.

Medication abortion practice in the US and Canada follows evidence-based guidelines. Best practice for analgesics and prophylactic antibiotics require further research. Concerted efforts to update practice based on the latest evidence for reducing in-person visits and increasing the ability of advanced practice clinicians to provide medication abortions could strengthen these services and reduce access barriers.
